# The Second Round of the PHAR-QA Survey of Competences for Pharmacy Practice

**DOI:** 10.3390/pharmacy4030027

**Published:** 2016-09-21

**Authors:** Jeffrey Atkinson, Kristien De Paepe, Antonio Sánchez Pozo, Dimitrios Rekkas, Daisy Volmer, Jouni Hirvonen, Borut Bozic, Agnieska Skowron, Constantin Mircioiu, Annie Marcincal, Andries Koster, Keith Wilson, Chris van Schravendijk

**Affiliations:** 1 Pharmacology Department, Lorraine University, 5 Rue Albert Lebrun, Nancy 54000, France; 2 Pharmacology Consultants Nancy, 12 rue de Versigny, Villers 54600, France; 3 Department of Pharmaceutical and Pharmacological Sciences, Vrije Universiteit Brussel, Laarbeeklaan 103, Brussels 1090, Belgium; kdepaepe@vub.ac.be; 4 Faculty of Pharmacy, University of Granada, Campus Universitario de la Cartuja s/n, Granada 18701, Spain; sanchezp@ugr.es; 5 School of Pharmacy, National and Kapodistrian University Athens, Panepistimiou 30, Athens 10679, Greece; rekkas@pharm.uoa.gr; 6 Institute of Pharmacy, Faculty of Medicine, University of Tartu, Nooruse 1, Tartu 50411, Estonia; daisy.volmer@ut.ee; 7 Pharmacy Faculty, University of Helsinki, Yliopistonkatu 4, P.O. Box 33-4, Helsinki 00014, Finland; jouni.hirvonen@helsinki.fi; 8 Faculty of Pharmacy, University of Ljubljana, Askerceva Cesta 7, Ljubljana 1000, Slovenia; Borut.Bozic@ffa.uni-lj.si; 9 Pharmacy Faculty, Jagiellonian University, Golebia 24, Krakow 31-007, Poland; askowron@cm-uj.krakow.pl; 10 Pharmacy Faculty, University of Medicine and Pharmacy “Carol Davila” Bucharest, Dionisie Lupu 37, Bucharest 020021, Romania; constantin.mircioiu@yahoo.com; 11 Faculty of Pharmacy, European Association of Faculties of Pharmacy, Université de Lille 2, Lille 59000, France; annie.marcincal@pharma.univ-lille2.fr; 12 Department of Pharmaceutical Sciences, European Association of Faculties of Pharmacy, Utrecht University, P.O. Box 80082, Utrecht 3508 TB, The Netherlands; A.S.Koster@uu.nl; 13 School of Life and Health Sciences, Aston University, Birmingham B47ET, UK; k.a.wilson@aston.ac.uk; 14 Medical Faculty, Vrije Universiteit Brussel, Laarbeeklaan 103, Brussels 1090, Belgium; chrisvs@vub.ac.be

**Keywords:** pharmacy, education, competences, framework, practice

## Abstract

This paper presents the results of the second European Delphi round on the ranking of competences for pharmacy practice and compares these data to those of the first round already published. A comparison of the numbers of respondents, distribution by age group, country of residence, etc., shows that whilst the student population of respondents changed from Round 1 to 2, the populations of the professional groups (community, hospital and industrial pharmacists, pharmacists in other occupations and academics) were more stable. Results are given for the consensus of ranking and the scores of ranking of 50 competences for pharmacy practice. This two-stage, large-scale Delphi process harmonized and validated the Quality Assurance in European Pharmacy Education and Training (PHAR-QA) framework and ensured the adoption by the pharmacy profession of a framework proposed by the academic pharmacy community. The process of evaluation and validation of ranking of competences by the pharmacy profession is now complete, and the PHAR-QA consortium will now put forward a definitive PHAR-QA framework of competences for pharmacy practice.

## 1. Introduction

PHAR-QA “Quality Assurance in European Pharmacy Education and Training”, funded by the European Commission, is producing a framework of competences for pharmacy practice [1] in line with the EU directive on sectoral professions and taking into account the diversity of the pharmacy profession and the on-going changes in healthcare systems and in the pharmaceutical industry. PHAR-QA asked academia, students and practicing pharmacists to rank competences for practice. The results of the first Delphi [2] round show that competences in the areas of “drug interactions”, “need for drug treatment” and “provision of information and service” ranked highest. This paper presents the results of the second PHAR-QA Delphi round in the European pharmacy community. A revised version of the PHAR-QA questionnaire was produced following the analysis of the results of the first round [2]. The expert academic panel (the authors) based their revision of the first European Delphi questionnaire on the ranking of, and comments on, the competences proposed. In most cases, the subject matter of the competences was unaltered in the second round survey compared to that of the first round.

Several problems were encountered in the first round [2], and changes were made in order to make the questionnaire clearer. The major changes in the revised version of the survey questionnaire were:

- Questions were simplified, especially regarding matters of:
  . treating one topic per question;
  . simplifying English expressions.
- The section on the subject areas as given in the directive 2013/55/EU (physics, biology, etc.) [3] was removed as these were not considered as “competences”.
- Questions on research and industrial pharmacy were reworked given the level for which the PHAR-QA framework is intended: five-year pharmacy degree, not postgraduate specialisation.
- Emphasis was placed on “being aware of”, rather than “capable of doing”. We used the terms “knowledge”, i.e., “being aware of”, and “ability”, i.e., “capable of doing”.
- The second version of the European Delphi questionnaire included an open-ended question for suggestions on matters not proposed that should be treated and other comments.

As the subject area of each competence was not altered, it is possible to compare the rankings of the second round with those of the first.

The rationality behind the study was double. Firstly, pharmacy departments need tools for implementation, but also for the evaluation of programs, striking a balance between a structural approach (subjects, years, etc.) and a competence approach (expected by patients and/or employers). Secondly, following the enormous development and dispersion of pharmacists’ professional activities, a diploma without additional information is not enough. This is not a question for the pharmacy degree alone, but is in line with increased expectations in terms of quality, usefulness and employability of graduates [4]. The rationality behind the use of the Delphi methodology was to determine a reliable group opinion from a group of experts with a measure of their consensus [5].

## 2. Experimental Section

### Short Description of the Experimental Paradigm and Methodology

The methodology/paradigm used in the PHAR-QA project has been described in detail elsewhere [2]. A summary is given in Table 1.

Each of the individual 3771 entries into the two rounds of the survey was analysed in detail. Entries from respondents not going beyond the first 6 questions (on the respondent profile) were removed, leaving a total of 2773 complete entries for the two rounds, Round 1, 1245, and Round 2, 1528 complete entries.

Several strategies were used to minimize bias. The small expert panel examined the formulation of questions in order to avoid “leading questions” involving suggestive interrogation evoking a particular answer from a particular group. Other biases could arise in the way in which respondents were approached. This has been described in detail elsewhere [2]. In order to avoid bias from a partial response ratio, we defined representative groups and did not send the questionnaire to general populations. However, this by itself could have introduced a bias. It is possible, for instance, that as we used national student associations to contact students (amongst other means) rather than sending the questionnaire to global listings of students, then we harvested results from students motivated to join a student union. The counter argument here is that such students may well be the ones interested in change and evolution in pharmacy education and training with, for instance, more competency-based education. A more general point here is that there may well be self-selection bias by respondents themselves with selection of those more concerned with the future of pharmacy. This may be desirable if the purpose of the Delphi procedure is to direct future developments rather than to confirm present opinions.

“Double replies” were defined as those of respondents with complete replies to the two surveys, separated in time by at least 9 months, both from the same computer Internet Protocol address (IP address) and having identical replies to the first 6 profile questions (age, profession, etc.) of the questionnaire. It was assumed that if the IP address and the replies to the first 6 questions were identical in the two rounds, then the same person was involved in the two rounds. There was no possibility to validate or invalidate this supposition.

## 3. Results and Discussion

### 3.1. Presentation of the Results of the Second Round in the European Pharmacy Community and Comparison with the Results of the First Round

The numbers of respondents in the two rounds are presented in Table 2. Compared to the first round, the percentage of respondents going beyond Question 6 in the second round was lower in all groups excepting that of “pharmacists in other professions”, where the response rate was higher in the second round (81% versus 48%).

The number of respondents in all groups and in both rounds was higher than the minimum number required to be surveyed based on the estimated European population of each group [2,13].

The percentage of double replies was generally low. It was highest for industrial (16%) and hospital pharmacists (15%; Table 2). Thus, in these two groups, just under one fifth of replies in the two rounds, in all probability, came from the same person. The percentage of double replies for students was very low (0.6%), showing that two different student populations answered the questionnaires in the two different rounds. This reveals a certain conflict in the student group between two of the principles of the Delphi methodology: iteration and anonymity. Iteration would require that the composition of the expert panel remains the same, i.e., that the same people are surveyed, in the different rounds. However, in order to maintain anonymity, the decision was taken not to collect email addresses in the first round and to resend the survey to the same email addresses in the second round (so ensuring that the same people were contacted). We hoped to overcome the difficulty arising from the conflict of “iteration versus anonymity” by using the same global email listings for the different groups in the two rounds and this with the objective of contacting the same people.

**Figure 1:**
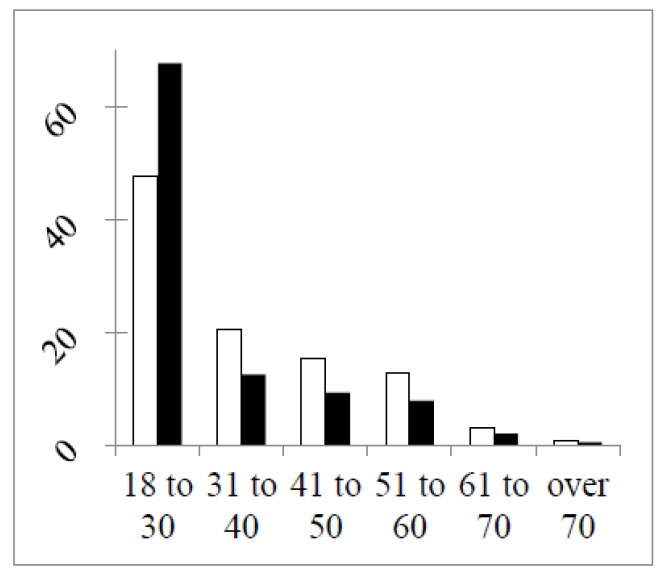
Distributions of respondents by age (%) in the two rounds (Round 1 open columns, Round 2 full columns). The chi-square test of a difference between rounds (df = 5, six groups): 16.8, *p* < 0.01. Chi-square (df = 4; without the student group): 0.6, *p* > 0.05.

In Figure 1 are shown the distributions of respondents by age in the two rounds.

**Figure 2:**
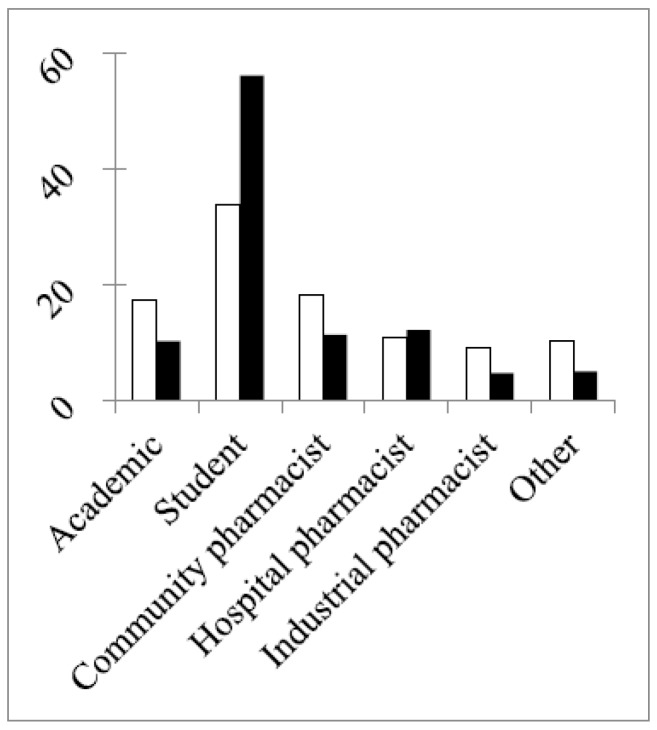
Percentage distribution of different groups in the two rounds (“other”: pharmacists working in other professions). The chi-square of a difference between rounds (df = 5, six groups): 13.2, *p* < 0.05. Chi-square (df = 4; without the student group): 2.8, *p* >0.05.

**Figure 3:**
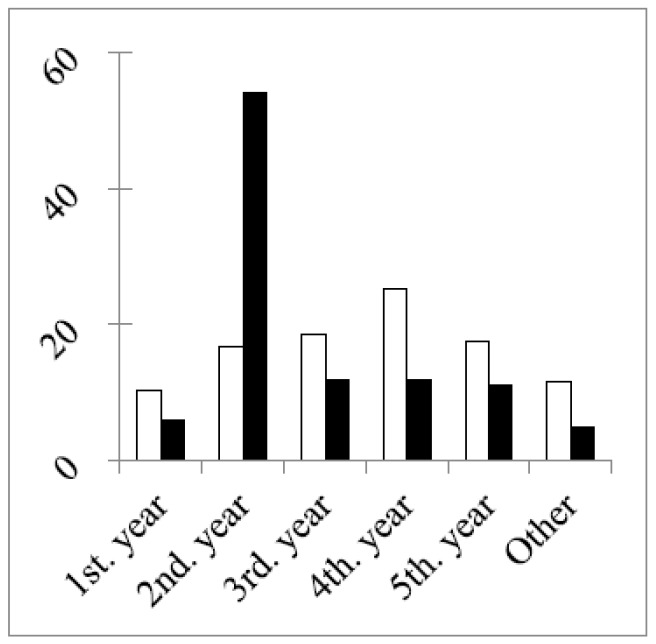
Study years of students (expressed as the % of the total in each round). The chi-square of a difference between rounds (df = 5, six groups): 32.3, *p* < 0.001. Chi-square (df = 4; without the second year group): 0.8, *p* > 0.05.

In both rounds, 50% or more of the respondents were in the age group 18–30 years old. This reached 68% in the second round due to a much larger percentage of students in this round (Figure 2) and to the difference in the study year of the students between the two rounds (Figure 3).

**Figure 4:**
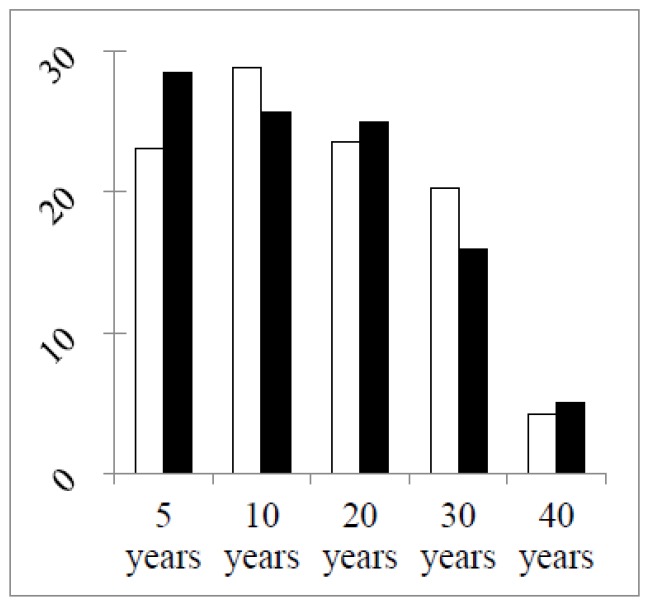
Percentage distributions of the years of professional experience of groups (excepting students). Chi-square (df = 4, five groups): 1.6, *p* > 0.05.

Figure 3 shows that the main difference in numbers concerned second year students, with almost four-times more students in the second year group in the second round. In other groups the total numbers and the numbers of double replies in the two rounds were similar (Table 2). The greater stability of the groups (excepting students) between the two grounds was illustrated by two other factors. Firstly, the percentage distributions of years of professional experience were similar in the two rounds (Figure 4).

The second factor revealing that the populations of professional groups were similar in the two rounds was the existence of a significant correlation between the numbers of respondents per country in the two rounds in three out of five of the groups: community, hospital and industrial pharmacists (Table 3). Thus, for these three groups, countries returned similar numbers of respondents in the two rounds.

**Figure 5:**
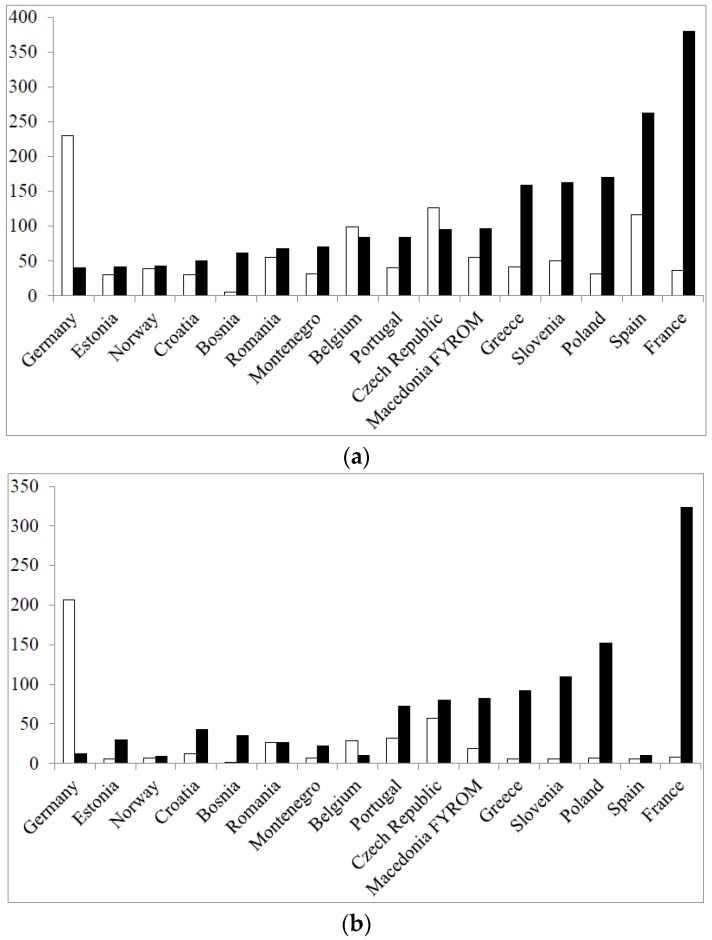
Total number of respondents per country (**a**); and number of students per country in the two rounds (**b**) (open columns: Round 1; full columns: Round 2).

The number of students per country in the two rounds determined to a large extent the total number of respondents per country (Figure 5) (data for the top 16 in terms of total respondents/country in Round 2 are given).

It can be seen that the large numbers of total respondents in the second round in countries such as France and Poland, for instance, were largely due to the recruitment of large numbers of students in these countries in Round 2. In other countries, e.g., Spain, this was not the case. Thus, although Table 3 reveals that for professional pharmacist groups, the geographical distribution of the respondent population is more or less stable from Round 1 to Round 2, Table 3 and Figure 5 reveal that for other groups, such as students and academics, this is less evident. Two questions have to be answered here: why is there a shift in geographical distribution of respondents in some groups, and does this impact on the global results? The methodology used was the same in both rounds and for all groups. It was essentially based on contacts by email, mainly via professional groups, chambers and associations, backed up by oral contact with individuals and groups. The same email lists were used in the two rounds. Albeit that although the same persons were contacted, the response rate was different, in some groups, from Round 1 to Round 2. Personal contacts suggested that this may have been due to different awareness of the project through national and European publications and to a fall in interest following surveying “fatigue”. Although the geographical distribution of respondents changed, this did not appear to modify the correlation between scores for individual competences obtained in the first round and those obtained in the second round (see later). This could be due to the fact that ranking by a given group changes little from one country to another [14].

### 3.2. Results for the Ranking of Competences and for Consensus in the Second Round

Overall ranking data are given in Table 4.

As in the first round, the total for “cannot rank” plus “blanks” was low (14.5%), suggesting that the questionnaire was easy to understand and relevant to practice. The calculated score for the total population (*n* = 1528 respondents) was high at 81.8%, revealing that globally, 8/10s of respondents ranked the competences as “obligatory”. The global Leik consensus was also high at 0.61, revealing that opinions were relatively homogeneous. These values are not significantly different from those of Round 1.

**Figure 6:**
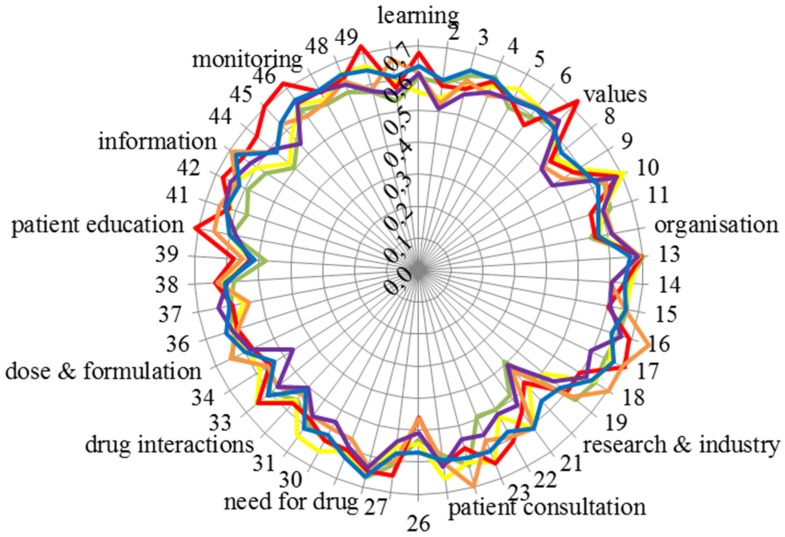
Leik consensus values for the 50 competences of the six groups (vertical scale: Leik consensus, circumference: competence number and cluster; community pharmacists: green; hospital pharmacists: orange; industrial pharmacists: red; pharmacists in other professions: purple; students: blue; academics: yellow). See the Appendix for the details of competences.

The Leik consensus values for the 50 competences for each of the six groups are given in Figure 6.

Consensus within groups was high (around 0.6) and similar for all groups. Consensus was relatively lower (0.4–0.5) for Competences 20 “knowledge of design, synthesis, isolation, characterisation and biological evaluation of active substances” and 26 “ability to perform appropriate diagnostic tests, e.g., measurement of blood pressure or blood sugar”.

Consideration of the Leik consensus values brings us back to the question of bias (which has already been touched upon (see above)). In order to avoid bias in selection, the numbers of respondents were planned to be balanced between countries and professional groups. Since the calculated Leik ordinal consensus between fractions in all groups was high, we considered that this was achieved and that the replies were homogeneous. On a more general basis, in a study such as this, it is unavoidable that certain biases will be present, and this is a possible limitation of the study. The two main elements here are the Delphi approach and the selection of experts. We would argue that the Delphi approach used allowed us to establish an expert group opinion with an acceptable degree of dispersion or consensus. The second element is our definition of experts, i.e., the groups approached for answering the questionnaire. On this point, some may disagree with our choice. Only future studies and data can provide an answer to this question.

**Figure 7:**
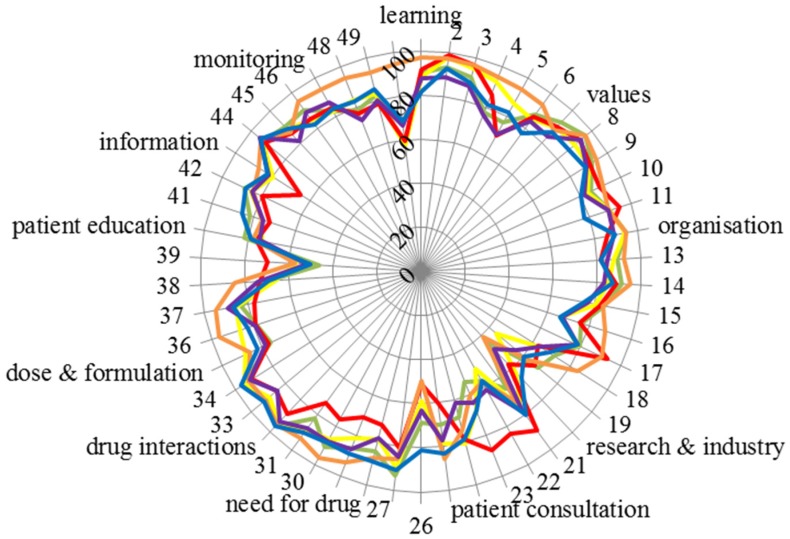
Scores for the 50 competences of the six groups (vertical scale: score, circumference: competence number and cluster; community pharmacists: green; hospital pharmacists: orange; industrial pharmacists: red; pharmacists in other professions: purple; students: blue; academics: yellow). See the Appendix for the details of competences.

The scores for the 50 competences of the six groups are given in Figure 7.

Figure 7 emphasises the consensus of scoring amongst groups. Scores were almost exclusively very high (80% or above) except for four competences: 20: “knowledge of design, synthesis, isolation, characterisation and biological evaluation of active substances”, 26: “ability to perform appropriate diagnostic tests, e.g., measurement of blood pressure or blood sugar”, 39: “ability to manufacture medicinal products that are not commercially available” and 50: “ability to contribute to the cost effectiveness of treatment by collection and analysis of data on medicines’ use” (see also the table in the Appendix). It should be noted that there was some disagreement within groups in scoring, as, for instance, Leik consensus on Competences 20 and 26 was low (Figure 6).

Another indicator of global consensus was the Spearman correlation coefficient. When compared with the community pharmacist, groups’ score values for the different groups were as follows: hospital pharmacists r = 0.62, industrial pharmacists r = 0.64, pharmacists in other professions r = 0.87, students r = 0.77 and academics r = 0.82 (all *p* < 0.001).

The above correlations are interesting in light of the change in the student contribution from the first to the second round. The proportion of students involved increased very substantially from under 30% (Round 1) to approximately 50% (Round 2). Furthermore, many of those students who responded were only in their second year of pharmacy education. These students may be unqualified to reliably answer such a survey, as they are some distance from actually working as a pharmacist. However the Spearman correlation coefficient for scores for students against scores for community pharmacists is high (0.77) and incidentally higher than that for hospital pharmacists (0.62). Visual inspection of Figure 7 shows that there is a tight relationship between scores for community pharmacists (green line) and students (blue line). Two other points should be considered. In a previous paper on the results from Round 1 [15], students, academics and community pharmacists ranked personal and patient care competences for pharmacy practice. The ranking profiles for all three groups were similar. This was true of the comparison between students and community pharmacists concerning patient care competences, suggesting that students do have a good idea of their future profession. Albeit, a comparison of first and fifth (final) year students did show slightly more awareness of patient care competences in the final year students. On balance, we would suggest that pharmacy students, even those in the early years of study, do have well-founded ideas on the competences required for their future profession. The same paper showed that there were no substantial differences amongst rankings of students from different countries, some with more “medicinal/clinical” courses and others with more “chemical sciences” courses. Secondly, in the PHARMINE study [16], it was found that 9/25 countries provide a substantial part of their training (community and/or hospital pharmacy) in the first two years of study. For example, second year French pharmacy students, who were largely represented in the second round, have a six-week training period.

There were some differences amongst groups, with for instance, hospital pharmacists scoring competence 50 “ability to contribute to the cost effectiveness of treatment by collection and analysis of data on medicines’ use” much higher (95%) than the overall average (68%). Another example was industrial pharmacists who score competences in Cluster 10 “research and industrial pharmacy” higher, often substantially, than the global average (see the table in the Appendix).

**Figure 8:**
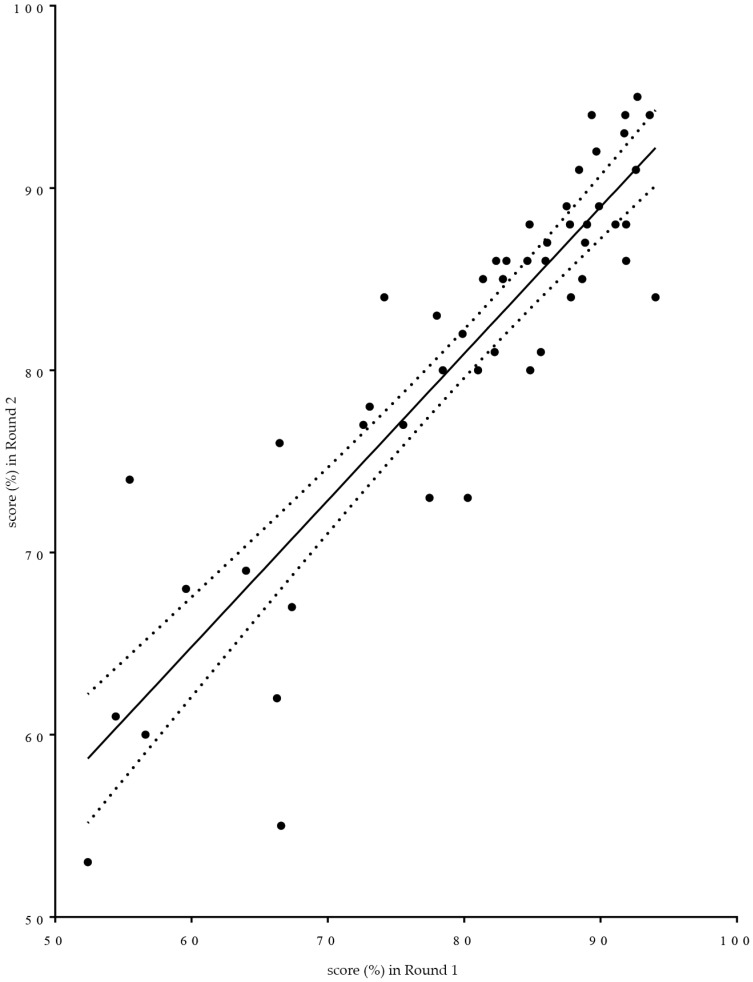
Linear regression graphic representation of the relationship between scores for individual competences obtained in the first round of the PHAR-QA European Delphi survey (*x* axis) and those obtained in the second round (*y* axis) (dotted lines: 95% confidence interval).

Given that the subject matter of proposed competences was not substantially altered between rounds, scores for the same competences were compared between Rounds 1 and 2. Linear regression analysis was used (see Figure 8). Two provisos have to be made regarding such use. It is not known whether the variable “score” is normally distributed. Given that the score is a transformed variable calculated on the basis of ranks that are highly skewed to the right suggests that scores may have a non-normal distribution. Furthermore, the exact wording of the individual questions asked differed between the two rounds. In Figure 8 is given as a graphic aid to understanding of the relationships between the two rounds. The non-parametric Spearman correlation coefficient “r” was 0.88, *p* < 0.0001. Global means were 80% for Round 1 and 81% for Round 2.

In spite of the fact that, in the second round compared to the first, not exactly the same population was questioned and not exactly the same questions were asked, rankings were similar.

The numbers of comments are shown in the Table 5.

The number of comments was low (3% in the global population), as in the first Delphi study [17]; there were on average two comments per commentator. There were no suggestions as to topics that were not, but should have been, included.

As in Round 1, comments were mainly (31/113) on four topics:

(1) Working environment (six comments). Example: *emphasis should be put on the community pharmacy and hospital pharmacy setting*.
(2) Team work and the definition of the responsibility of the pharmacists within the health team (14 comments). Example: *clearly know what the pharmacist is responsible for*. One community pharmacist suggested that pharmacists were ideally suited to be the “coordinator” of the health team.
(3) Legal and other limits to the pharmacist’s responsibility (seven comments). Examples: *diagnosis is the responsibility of doctors; pharmacists in Latvia mostly work in chain-pharmacies…where owners and managers have no pharmaceutical education*.
(4) Use of information technology (four comments). Example: *ability to find appropriate sources and use electronic platforms*.

The other 82 comments were on very diverse topics. This prevented the evaluation of any definitive pattern in comments with the use of semi-quantitative data analysis software.

## 4. Conclusions

The essence of the methodology of the PHAR-QA approach can be summarized as follows. We started with a framework based on PHARMINE [6]. To this, we added elements from frameworks used by other healthcare professions, doctors [9] and dentists [18]. Finally, frameworks used in other countries, such as the U.K. [19], Canada [20] and Australia [21], for pharmacists were used. This was refined by three Delphi rounds within a small expert panel (authors of this paper). This approach has been used in previous studies and can be criticised on the basis that there is no real “transfer” of the framework from academics to practitioners. Such a transfer was attempted in the MEDINE study in which the framework proposed by the academic community passed through a (single) Delphi round within a large expert panel consisting of European medical practitioners and students. In PHAR-QA, such validation was taken further by having two Delphi rounds with a large expert panel of European pharmacy professionals, academics and students. The aim of this two-round approach was to harmonise, as well as validate the competence framework and ensure that a framework elaborated by an academic expert panel would be adopted by the European pharmacy community.

The question can be asked as to whether such a “double Delphi” process works. There are several indications that this is the case. Firstly, the ranks given varied widely from 41%–99%. Respondents did not give a “global” rank that was more or less the same for all competences, and yet, secondly, there was good consensus within the groups and amongst the groups. Thirdly, except for students, there were a significant number of double replies. Fourthly, again, except for students, the profiles of the responding groups in terms of age, country of origin, years of experience, etc., were similar in the two rounds. Thus although, for the sake of anonymity, we did not collect email addresses in the first round and send the second version of the survey to the same email addresses, it would appear that in the professional groups, the profiles of respondents are similar in the two rounds. Fifthly, the replies obtained in the two rounds were highly correlated (see Figure 8).

One proviso on the statistical methodology used has to be added. On several occasions in this study, parametric statistics have been used for variables that may not necessarily follow a normal distribution. This was done in the analysis of the data of the first round [2] and justified on such occasions by the fact that parametric tests are robust [22].

Finally, a few observations on the use of the PHAR-QA competence framework are presented below:

- A good starting point for the adoption of the competence framework is to match existing curriculum of a department to the framework; this approach has previously been used to match outcomes to curricula, and the methodology has been published [23].
- Building a curriculum based on the PHAR-QA competence framework could be guided by the following:
  ○ Core curriculum: the fundamental, bachelor curriculum could be based, amongst others, on the clusters of competences with the highest ranking scores, *viz*, 12: “need for drug treatment”, 13: “drug interactions” and 16: “provision of information and service”.
  ○ Specialisation in the advanced, master curriculum could include, for instance,
    ◾ for community pharmacists: Competences 32 “ability to identify and prioritise drug-drug interactions and advise appropriate changes to medication” and 34 “ability to identify and prioritise drug-disease interactions (e.g., NSAIDs in heart failure) and advise on appropriate changes to medication”
    ◾ for hospital pharmacists: Competences 36 “ability to recommend interchangeability of drugs based on in-depth understanding and knowledge of bioequivalence, bio-similarity and therapeutic equivalence of drugs” and 50 “ability to contribute to the cost effectiveness of treatment by collection and analysis of data on medicines’ use”
    ◾ for industrial pharmacists; Competences 21 “knowledge of good manufacturing practice and of good laboratory practice” and 23 “knowledge of drug registration, licensing and marketing”
  ○ The ways in which the various competences are taught are diverse. For instance, for personal competence Clusters 8 “values” and 9 “communication and organisational skills”, the role of the traineeship monitor is uppermost. The way in which this is to be developed needs to be harmonized within the EU, but the finer details would be up to individual faculties.

The overall conclusion of the PHAR-QA study is that pharmacists, regardless of their career path, have a great deal in common; all are seeking for improvements in their services to patients. Their different perspectives in certain areas represent a solid starting point for steering the necessary changes through discussions within their professional organisations and faculties. PHAR-QA intended to bridge the different career paths through consensus; thus, all concerned can view these findings as an opportunity to further strengthen the scientific role of the pharmacist.

## Figures and Tables

**Table 1:** Research paradigm used. PHAR-QA, Quality Assurance in European Pharmacy Education and Training.

Step	Phase
1	A competence framework based on PHARMINE [6] and other published frameworks for practice in healthcare was ranked (4-point Likert scale) and refined by 3 rounds of a Delphi process [7], by a small expert panel consisting of the authors of this paper.
2	Following the 3rd Delphi round within the small expert panel above, the competences were ranked in two separate rounds by a large expert panel consisting of six groups, European academics, students and practicing pharmacists (community, hospital, industrial and pharmacists working in other professions), using the PHAR-QA SurveyMonkey^®^ (SurveyMonkey Company, Palo Alto, CA, USA) questionnaire [8]. There were 68 competences proposed in the first round and 50 in the second, the difference being due primarily to the removal of the subject areas. Invitations were sent to the 43 countries of the European Higher Education Area that have university pharmacy departments (thus excluding countries, such as Luxembourg and the Vatican). Data were obtained from 38 countries (thus not including Armenia, Azerbaijan, Georgia, Moldova and Russia). In some figures, not all countries are represented, but data from all countries were included in the statistical analysis.
3	The first 6 questions were on the profile of the respondent (age, occupation, experience).
4	Respondents were then asked to rank clusters of questions on competences numbered 7–17 (numbering following on from the 6th question of the respondent profile). Questions in Clusters 7 through 10 were on personal competences and in Clusters 11–17 on patient care competences.
5	Respondents were asked to rank the proposals for competences on a 4-point Likert scale:
	(1) Not important = Can be ignored;
	(2) Quite important = Valuable, but not obligatory;
	(3) Very important = Obligatory, with exceptions depending on the field of pharmacy practice;
	(4) Essential = Obligatory.
	There was also a “cannot rank” possibility and the possibility of leaving an answer blank.
6	Ranking scores were calculated (frequency rank 3 + frequency rank 4) as the % of total frequency; this represents the percentage of respondents that considered a given competence as “obligatory”.
	The calculation of scores is based on that used by the MEDINE “Medical Education in Europe” study [9].
7	Leik ordinal consensus [10] was calculated as an indication of the dispersion of the data within a given group. Responses for consensus were arbitrarily classified as: <0.2 poor, 0.21–0.4 fair, 0.41–0.6 moderate, 0.61–0.8 substantial, >0.81 good, as in the MEDINE study [7].
8	For differences amongst groups and amongst competences, the statistical significance of differences was estimated from the chi-square test; a significance level of 5% was chosen. Correlation was estimated from the non-parametric Spearman’s “r” coefficient and graphically represented using parametric linear regression.
9	Respondents could also comment on their ranking. An attempt was made to analyse comments using the NVivo10^®^ (QSR International Pty Ltd., Victoria, Australia) [11] and the Leximancer^®^ (Leximancer Pty Ltd., Brisbane, Australia) [12] programs for the analysis of semi-quantitative data. In this study and the previous first round study, the word number of the comments was too small to draw significant conclusions.

**Table 2:** The numbers of respondents in the two rounds.

	Round 1	February 2014–November 2014		Round 2	August 2015–February 2016		n of Double Replies **	Double Replies %
Group	Total Number of Web Entries	Respondents Going beyond Question 6 *	% Respondents Going beyond Question 6	Total Number of Web Entries	Respondents Going beyond Question 6 *	% Respondents Going beyond Question 6		
Community pharmacists	285	258	91	264	183	69	16	9
Industrial pharmacists	140	135	97	109	93	85	15	16
Hospital pharmacists	173	152	88	271	188	69	29	15
Pharmacists in other professions	159	77	48	89	72	81	4	5
Students	529	382	72	1250	785	63	5	0.6
Academics	267	241	90	235	207	88	21	10
*Total*	*1553*	*1245*	*NA*	*2218*	*1528*	*NA*	*NA*	*NA*
*Average*	*NA*	*NA*	*80*	*NA*	*NA*	*69*	*NA*	*9*

* The first 6 questions were on profile (age, profession, etc.). The first 6 “profile” questions were identical in the 2 rounds of the survey. ** “Double replies” are defined as those of respondents with complete replies to the two surveys, separated in time by at least 9 months, both from the same computer Internet Protocol address (IP address) and having identical replies to the first 6 profile questions (age, profession, etc.) of the questionnaire.

**Table 3:** Correlation (r) between the numbers of respondents per country in the two rounds. NS: *p* > 0.05.

Group	r	*p*
Community pharmacists	0.24	*p* < 0.05
Hospital pharmacists	0.68	*p* < 0.05
Industrial pharmacists	0.15	*p* < 0.05
Pharmacists in other professions	0.00002	NS
Academics	0.02	NS
Students	0.0007	NS

**Table 4:** Ranking data for the total population in the second round (*n* = 1528 respondents).

Ranking	Number of Rankings	%
Essential	25,426	33.3
Very important	27,959	36.6
Quite important	10,708	14.0
Not important	1240	1.6
Cannot rank	1909	2.5
*Subtotal*	*67,242*	*88.0*
Blanks	9158	12.0
*Total*	*1528 × 50 = 76,400*	*100.0*

**Table 5:** Numbers of commentators and comments.

Group	Number of Respondents	Number of Commentators	% Respondents Commenting	Number of Comments	Number of Comments/Commentator
Community pharmacists	183	6	3.3	19	3.17
Hospital pharmacists	188	8	4.3	13	1.63
Industrial pharmacists	93	3	3.2	13	4.33
Pharmacists working in other professions	72	6	8.3	8	1.33
Students	785	16	2.0	33	2.06
Academics	207	11	5.3	27	2.45
*Total*	*1528*	*50*	*3.3*	*113*	*2.26*

## Appendix A.

**Table A1:** Ranking of Competences.

Question	Competence	Community	Industrial	Hospital	Others	Students	Academics	Mean
	7. Personal competences: learning and knowledge.							88
**1**	**1. Ability to identify learning needs and to learn independently (including continuous professional development (CPD)).**	**90**	**91**	**97**	**87**	**82**	**88**	**89**
**2**	**2. Ability to apply logic to problem solving.**	**93**	**99**	**97**	**89**	**93**	**98**	**95**
**3**	**3. Ability to critically appraise relevant knowledge and to summarise the key points.**	**91**	**97**	**97**	**87**	**88**	**95**	**92**
**4**	**4. Ability to evaluate scientific data in line with current scientific and technological knowledge.**	**78**	**86**	**95**	**76**	**81**	**93**	**85**
**5**	**5. Ability to apply preclinical and clinical evidence-based medical science to pharmaceutical practice.**	**77**	**70**	**94**	**71**	**82**	**87**	**80**
**6**	**6. Ability to apply current knowledge of relevant legislation and codes of pharmacy practice.**	**88**	**87**	**94**	**84**	**77**	**85**	**86**
	8. Personal competences: values.							89
**7**	**1. A professional approach to tasks and human relations.**	**93**	**90**	**88**	**84**	**87**	**89**	**88**
**8**	**2. Ability to maintain confidentiality.**	**96**	**93**	**97**	**94**	**87**	**90**	**93**
**9**	**3. Ability to take full responsibility for patient care.**	**92**	**90**	**94**	**89**	**88**	**90**	**91**
**10**	**4. Ability to inspire the confidence of others in one's actions and advice.**	**85**	**90**	**91**	**82**	**80**	**83**	**85**
**11**	**5. Knowledge of appropriate legislation and of ethics.**	**90**	**94**	**90**	**89**	**78**	**90**	**88**
	9. Personal competences: communication and organisational skills.							79
**12**	**1. Ability to communicate effectively** **, both oral and written, in the locally relevant language.**	**95**	**86**	**94**	**89**	**89**	**94**	**91**
**13**	**2. Ability to effectively use information technology.**	**89**	**81**	**92**	**84**	**82**	**88**	**86**
**14**	**3. Ability to work effectively as part of a team.**	**91**	**88**	**95**	**83**	**87**	**85**	**88**
**15**	**4. Ability to implement general legal requirements that impact upon the practice of pharmacy (e.g., health and safety legislation, employment law).**	**84**	**83**	**84**	**77**	**74**	**80**	**80**
16	5. Ability to contribute to the training of staff.	76	76	88	67	66	67	73
**17**	**6. Ability to manage risk and quality of service issues.**	**80**	**93**	**90**	**78**	**78**	**79**	**83**
18	7. Ability to identify the need for new services.	72	63	84	63	69	63	69
19	8. Ability to understand a business environment and develop entrepreneurship.	69	67	63	56	60	44	60
	10. Personal competences: research and industrial pharmacy.							66
20	1. Knowledge of design, synthesis, isolation, characterisation and biological evaluation of active substances.	44	58	41	48	66	63	53
21	2. Knowledge of good manufacturing practice and of good laboratory practice.	65	89	73	78	81	76	77
22	3. Knowledge of European directives on qualified persons.	56	84	57	61	57	51	61
23	4. Knowledge of drug registration, licensing and marketing.	54	87	60	64	68	67	67
24	5. Knowledge of the importance of research in pharmaceutical development and practice.	68	78	74	62	78	81	74
	11. Patient care competences: patient consultation and assessment.							75
25	1. Ability to interpret basic medical laboratory tests.	70	60	86	77	83	79	76
26	2. Ability to perform appropriate diagnostic tests, e.g., measurement of blood pressure or blood sugar.	69	51	49	63	81	59	62
**27**	**3. Ability to recognise when referral to another member of the healthcare team is needed.**	**93**	**81**	**86**	**84**	**91**	**90**	**87**
	12. Patient care competences: need for drug treatment.							85
**28**	**1. Ability to retrieve and interpret information on the patient’s clinical background.**	**84**	**72**	**87**	**78**	**89**	**78**	**81**
**29**	**2. Ability to compile and interpret a comprehensive drug history for an individual patient.**	**86**	**71**	**93**	**86**	**89**	**81**	**84**
**30**	**3. Ability to identify non-adherence to medicine therapy and make an appropriate intervention.**	**91**	**76**	**96**	**88**	**89**	**87**	**88**
**31**	**4. Ability to advise to physicians on the appropriateness of prescribed medicines and** **, in some cases, to prescribe medication.**	**83**	**74**	**93**	**88**	**90**	**89**	**86**
	13. Patient care competences: drug interactions.							92
**32**	**1. Ability to identify and prioritise drug-drug interactions and advise appropriate changes to medication.**	**96**	**89**	**95**	**94**	**96**	**94**	**94**
**33**	**2. Ability to identify and prioritise drug-patient interactions, including those that prevent or require the use of a specific drug, based on pharmaco-genetics, and advise on appropriate changes to medication.**	**90**	**85**	**92**	**85**	**92**	**89**	**89**
**34**	**3. Ability to identify and prioritise drug-disease interactions (e.g., NSAIDs in heart failure) and advise on appropriate changes to medication.**	**96**	**91**	**93**	**92**	**97**	**95**	**94**
	14. Patient care competences: drug dose and formulation.							76
**35**	**1. Knowledge of the bio-pharmaceutical, pharmacodynamic and pharmacokinetic activity of a substance in the body.**	**75**	**76**	**86**	**78**	**82**	**88**	**81**
**36**	**2. Ability to recommend interchangeability of drugs based on in-depth understanding and knowledge of bioequivalence, bio-similarity and therapeutic equivalence of drugs.**	**79**	**79**	**96**	**79**	**83**	**86**	**84**
**37**	**3. Ability to undertake a critical evaluation of a prescription ensuring that it is clinically appropriate and legally valid.**	**83**	**77**	**95**	**89**	**87**	**86**	**86**
38	4. Knowledge of the supply chain of medicines thus ensuring timely flow of quality drug products to the patient.	69	73	85	75	69	66	73
39	5. Ability to manufacture medicinal products that are not commercially available.	46	70	57	52	51	57	55
	15. Patient care competences: patient education.							80
40	1. Ability to promote public health in collaboration with other professionals within the healthcare system.	82	76	78	75	79	73	77
41	2. Ability to provide appropriate lifestyle advice to improve patient outcomes (e.g., advice on smoking, obesity, etc.).	81	72	76	75	85	76	78
**42**	**3. Ability to use pharmaceutical knowledge and provide evidence-based advice on public health issues involving medicines.**	**89**	**80**	**84**	**85**	**88**	**85**	**85**
	16. Patient care competences: provision of information and service.							87
**43**	**1. Ability to use effective consultations to identify the patient's need for information.**	**82**	**65**	**87**	**82**	**82**	**79**	**80**
**44**	**2. Ability to provide accurate and appropriate information on prescription medicines.**	**93**	**92**	**94**	**95**	**94**	**94**	**94**
**45**	**3. Ability to provide evidence-based support for patients in selection and use of non-prescription medicines.**	**90**	**86**	**87**	**81**	**88**	**89**	**87**
	17. Patient care competences: monitoring of drug therapy.							81
**46**	**1. Ability to identify and prioritise problems in the management of medicines in a timely and effective manner and so ensure patient safety.**	**91**	**86**	**95**	**89**	**82**	**85**	**88**
**47**	**2. Ability to monitor and report adverse drug events and adverse drug reactions (ADEs and ADRs) to all concerned, in a timely manner, and in accordance with current regulatory guidelines on good pharmacovigilance practices (GVPs).**	**83**	**85**	**94**	**87**	**84**	**83**	**86**
**48**	**3. Ability to undertake a critical evaluation of prescribed medicines to confirm that current clinical guidelines are appropriately applied.**	**78**	**77**	**94**	**74**	**83**	**83**	**82**
**49**	**4. Ability to monitor patient care outcomes to optimise treatment in collaboration with the prescriber.**	**83**	**78**	**93**	**80**	**85**	**83**	**84**
50	5. Ability to contribute to the cost effectiveness of treatment by collection and analysis of data on medicines’ use.	58	59	95	67	69	58	68

Ranking scores were calculated (frequency rank 3 + frequency rank 4) as % of total frequency; this represents the percentage of respondents that considered a given competence as “obligatory”. Competences in bold are those receiving a mean score >80%, i.e., 8/10 respondents considered these competences as “obligatory”.
